# The impact of pandemic-related stress on attentional bias and anxiety in alexithymia during the COVID-19 pandemic

**DOI:** 10.1038/s41598-023-33326-5

**Published:** 2023-04-18

**Authors:** Shu-Hui Lee, Kuan-Te Lee

**Affiliations:** 1 Center for General Education, National Tsing Hua University, Hsinchu, Taiwan; 2 Department of Educational Psychology and Counseling, National Tsing Hua University, Hsinchu, Taiwan

**Keywords:** Psychology, Human behaviour

## Abstract

The COVID-19 pandemic had negative consequences for mental health, yet it is unknown how and to what extent the psychological outcomes of this stressful event are moderated by individual traits. Alexithymia is a risk factor for psychopathology, and thus likely predicted individual differences in resilience or vulnerability to stressful events during the pandemic. This study explored the moderating role of alexithymia in the relationships of pandemic-related stress with anxiety levels and attentional bias. The participants were 103 Taiwanese individuals who completed a survey during the outbreak of the Omicron wave. Additionally, an emotional Stroop task including pandemic-related or neutral stimuli was used to measure attentional bias. Our results demonstrate that pandemic-related stress had a lesser impact on anxiety in individuals with a higher level of alexithymia. Moreover, we found that in individuals with higher exposure to pandemic-related stressors, a higher level of alexithymia indicated less attentional bias toward COVID-19-related information. Thus, it is plausible that individuals with alexithymia tended to avoid pandemic-related information, which could temporarily relieve stressors during the pandemic.

## Introduction

The World Health Organization (WHO) declared the COVID-19 a pandemic in March 2020; thereafter, dramatic global spread occurred^1^. Currently, the number of infections and the case fatality rate continue to accumulate^2,3^. Compared to other countries, Taiwan was relatively resilient to the threat of COVID-19 due to acute awareness of the news about COVID-19 and rapid implementation of approaches to protect public health by the government^4,5^. However, the Omicron variant wave struck Taiwan in May 2022, and the psychological impacts were profound. Compared to previous waves, the Omicron variants were more contagious^6,7^, with breakthrough infections occurring even in individuals with multiple vaccine combinations^8^. With the greater concern over infection during this wave, it is plausible that psychological impacts such as frustration, irritability, burnout, and insomnia would become widespread in response to the prolonged restrictions of social isolation and quarantine practices^2^. Thus, it is vital to explore the psychological impact of COVID-19 on Taiwanese individuals during this wave.

Compared to the prepandemic period, psychological problems such as depression, sleep disorders, and posttraumatic stress symptoms (PTSS) have been frequently reported in the general population during the pandemic^9–12^. Recent studies have suggested that these psychological problems are associated with exposure to COVID-19-related stressors^13,14^, including social isolation, financial losses, and changes in interpersonal relationships, as well as health-related factors, such as uncertainty about the length of the pandemic process and fears of infection and its consequences^9,13,15^. These detrimental effects may hinder emotion regulation and the ability to attend to and retrieve information; moreover, pandemic-related restrictions can impact not only short-term but also long-term mental health^9^. These pandemic-related stressors can lead to psychological and physiological risks, such as anxiety, depression, poor physical condition and disrupted emotional and cognitive functioning^13,16–20^.

Psychological outcomes of exposure to stressful events are moderated by individual traits^21–24^. Reactions to pandemic-related stressors may vary across individuals^25^. It is plausible that some individuals are more susceptible to stress and more likely to experience mental health problems during lockdown^26,27^. Thus, identifying individual differences in resilience or vulnerability to stressful events is critical to promote well-being during the pandemic.

Individual traits such as alexithymia have been implicated as a modulator of the psychological outcomes^28^. Alexithymia is a multifaceted personality trait involving difficulty in identifying and expressing feelings and an externally oriented thinking style^29,30^; it is associated with stress-related disorders, including anxiety, PTSS and depression^31–33^. According to the stress-alexithymia hypothesis, a lack of emotional awareness in individuals with alexithymia may lead to maladaptive responses when encountering stressors, in turn increasing susceptibility to mental problems^34^. In support of this, empirical evidence has determined that alexithymia is a transdiagnostic risk factor for psychopathology^35–37^. For instance, research has shown that alexithymia moderates the association between traumatic experiences and PTSS^38,39^. Increased levels of anxiety have also been reported as a common psychological reaction to the COVID-19 pandemic^40–42^, thus receiving ample attention in psychopathological research. However, these studies have not considered the moderation effects of alexithymia. Considering that stress levels have increased dramatically during the COVID-19 pandemic^9^, it is important to further explore whether alexithymia moderates the association between COVID-19-related stressors and levels of anxiety.

Recent studies have also highlighted the importance of examining attentional bias in response to elevated levels of stress during COVID-19^17,43,44^. Specifically, attentional bias is defined as the tendency to pay attention to survival-related information, reflecting greater attentional allocation toward negative information than neutral information^45–47^. Recent studies have indicated that even mere exposure to negative information about COVID-19 (e.g., news or rumors about COVID-19) leads to increased levels of anxiety^48^. This negative information is distributed through various media platforms, making it impossible to ignore it in daily life. According to evolutionary accounts, negative information is better at capturing attention (i.e., signal detection of potential threats or contamination) and eliciting subsequent physiological and behavioral responses^49–51^. Negative stimuli may convey survival-related information, eliciting a defensive response to minimize the exposure to contamination (e.g., bodily fluids associated with infection or diseases)^50,52,53^.

Despite the necessity of effective detection of potential threats for survival, there are individual differences in attentional allocation toward threatening information^46,47,54^. In clinical research, attentional bias toward threat-related information is a robust phenomenon observed in anxious populations^45,55,56^. For instance, individuals with anxiety disorders showed faster response times toward threat-related stimuli than toward the neutral stimuli, reflecting hypervigilance toward potential harm^57,58^. Moreover, attentional bias toward threat-related information has been suggested as a crucial pathogenetic indicator for the etiology and maintenance of anxiety disorders^58,59^. However, this effect is not well established in alexithymia research^29^. Specifically, there is an unresolved debate regarding attentional bias and whether alexithymia causes disengagement difficulties (i.e., overresponding) or an early avoidance of processing threats^29,54,60^. Based upon this debate, two main hypotheses have been proposed. The overresponding hypothesis suggests that upon the detection of potential threats, individuals with a higher level of alexithymia may exhibit prolonged attention to unpleasant information versus neutral information^29,54^. This maintenance of attentional bias may be due to difficulty in regulating top-down attentional allocation for emotionally ambiguous situations, biasing attention in a threat-related manner and selecting negative (or less positive) interpretations of potential threats^61^. In response to this preattentional evaluation of threats, several studies have reported that individuals with alexithymia demonstrate hypervigilance and unnecessary depletion of attentional resources, resulting in difficulty in disengaging away from the threatening stimuli^62,63^. Conversely, according to the vigilance–avoidance hypothesis of attentional bias^57,64^, individuals with alexithymia may demonstrate early facilitation of orienting attention toward potential threats, which initiates their subsequent defensive reactions (i.e., early avoidance) for further processing the negative stimuli. It is proposed that this reflects an immediate defensive approach to downregulate intense subjective experiences^65,66^. In support of this notion, previous studies found that individuals with alexithymia exhibit less effort (i.e., faster response time) for processing negative information compared to neutral information, which is due to lower allocation of attentional resources toward emotional information^67–69^. Currently, the ecological manifestation of these attentional biases in the presence of real-life stressors has not been elucidated. Given these controversial results, such research is crucial to understand how individual differences in alexithymic traits promote vulnerability or resilience to stressful experiences during the COVID-19 pandemic. Thus, we explored whether alexithymic traits modulate the association between COVID-19-related stressors and attentional bias.

In this cross-sectional study, we aimed to investigate whether alexithymia contributes to vulnerability to anxiety levels in response to stressors related to the COVID-19 pandemic; we focused on the responses of Taiwanese college students to the Omicron wave, and assessments were conducted from May 10 to June 16, 2022. Several studies have investigated the role of alexithymia in the COVID-19 pandemic in both the general population and among health care workers^70–73^. Nevertheless, it remains unclear whether alexithymia is a potential risk factor for psychopathology during the COVID-19 pandemic among the college student population. Given that college students are more vulnerable to stress-related symptoms due to their lack of life experience and emotional instability^74,75^, we hypothesized that COVID-19-related stressors elevate levels of anxiety and that this effect is moderated by alexithymia. Our second objective was to examine attentional bias toward negative information about the COVID-19 pandemic. We proposed that negative information is better at attracting attention and at triggering either disengagement or early avoidance of threats^62,68^. Negative information may convey critical survival-related information during the pandemic, eliciting reactivity to minimize exposure to infection^50,52,53^. Thus, we hypothesized that pandemic-related stressors would influence attentional bias to negative information and that this effect would be moderated by alexithymia. This work is anticipated to provide empirical evidence of the role of alexithymia in psychopathological outcomes during the pandemic.

## Methods

### Participants

A total of 192 students accepted an invitation via social media to complete a battery of self-report questionnaires in an online survey. For inclusion, students had to meet the following inclusion criteria: (1) right-handed, (2) normal or corrected-to-normal vision, (3) normal hearing, and (4) free of psychiatric disorders, neurological diseases, or psychotropic medication use. To control for the influence of anxiety and depressive symptoms^28,29,76^, the 9-item Patient Health Questionnaire (PHQ-9) and the 7-item generalized anxiety disorder scale (GAD-7) were also conducted to screen for psychiatric disorders. Of these students, 62 students with scores of ten or greater on the PHQ-9 and GAD-7 were excluded^77–79^. Students were contacted again later during the pandemic; at this time, 27 students declined to participate in the experimental session, resulting in a final sample of 103 participants (65 females, *M*_age_ = 23.13, *SD*_age_ = 2.81). Informed consent was obtained from each participant. The informed consent procedures were approved by the Research Ethics Committee of the National Tsing-Hua University. The experimental procedures were carried out in accordance with the Declaration of Helsinki. All participants received reimbursement for their time and participation in the study (approximately 15 USD).

### Measures

#### Levels of alexithymia

The level of self-reported alexithymia was assessed using the Chinese version of the Toronto Alexithymia Scale (TAS-20-TW)^80^. The TAS-20 consists of 20 items rated on a 5-point Likert scale and features a three-factor structure: (1) difficulty identifying feelings (DIF), (2) difficulty describing feelings (DDF), and (3) externally oriented thinking (EOT). The stability of this factor structure has been confirmed in the Taiwanese population^80^. On the TAS-20, potential scores range from 20 to 100. Higher scores on this scale indicate a higher level of alexithymia. Regarding reliability, these items had satisfactory internal consistency (Cronbach’s *α* = 0.85).

#### Pandemic-related stress

Pandemic-related stress was measured using a 10-item self-report questionnaire adapted from Weissman^13^. These items mainly examine the extent to which participants’ lives were impacted by the COVID-19 pandemic (e.g., income, personal safety). Participants rate each item on a 5-point Likert scale ranging from 0 (“not at all”) to 4 (“extremely”). We utilized the total score (the sum of the 10 items) in the analysis. These items had satisfactory internal consistency (Cronbach’s *α* = 0.78).

#### Levels of state anxiety

Levels of state anxiety were measured using the Chinese version of the State-Trait Anxiety Inventory (C-STAI-S)^81^. In the state anxiety subscale, participants are asked to rate how they feel at the present moment (e.g., “I am tense”). It consists of 20 items rated on a 4-point Likert scale ranging from 0 (“not at all”) to 4 (“extremely”). Higher scores indicate higher levels of state anxiety. These items had satisfactory internal consistency (Cronbach’s *α* = 0.92).

#### Attentional bias

Attentional bias was measured using the emotional counting Stroop task^82,83^, which was presented using E-Prime software [version 3.0; Psychology Software Tools (PST)]. Participants took the computerized test in a quiet room. Before the formal test, there were six practice trials with feedback to ensure that participants understood the task; these items involved different stimuli than those used in the formal test. Each participant achieved at least 80% accuracy in each condition in the practice session.

**Figure 1:**
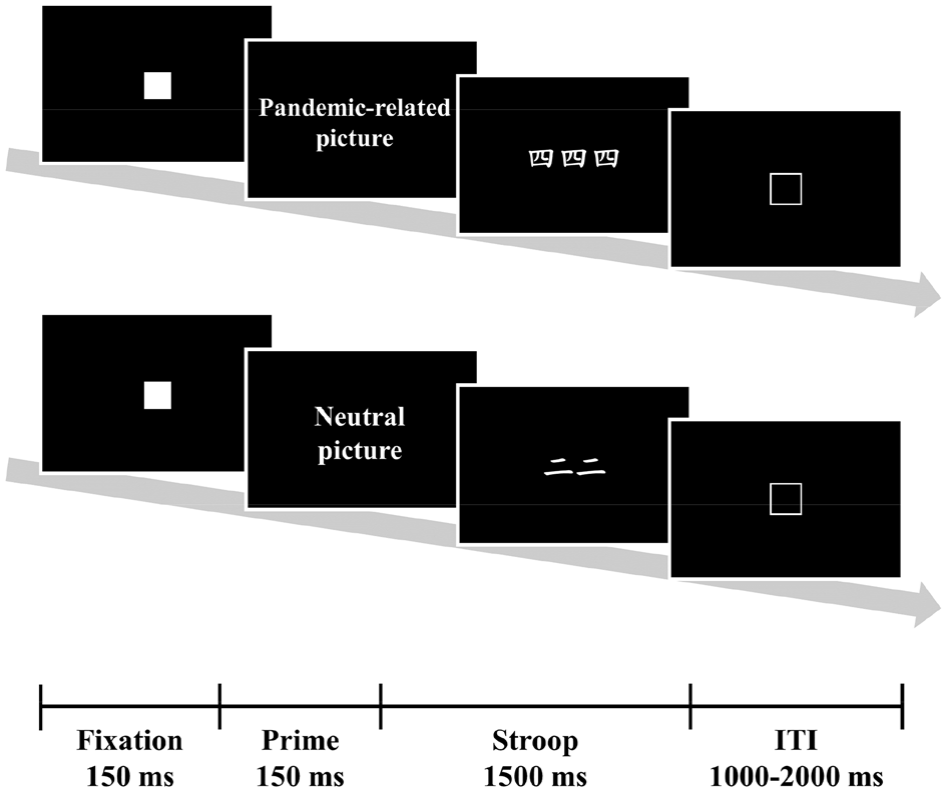
Experimental design of the emotional counting stroop task.

In the emotional counting Stroop task, participants were asked to perform a counting Stroop task preceded by emotional or neutral pictures. The task was categorized into congruent and incongruent conditions, with 60 trials each. For each of these conditions, trials were further divided into two emotional conditions, with 30 pandemic-related trials (e.g., images of pandemic-related objects, scenes of people getting vaccinated or people demonstrating COVID-19-related symptoms, scenes of social distancing behavior or people during lockdown) and 30 neutral trials (e.g., scenes of neutral objects, animals, or people with neutral expressions or in neutral situations). Additionally, 60 trials with either threatening or aversive pictures (e.g., scenes of aversive insects or threatening animals or scenes of people being threatened with weapons) were used as fillers in the congruent and incongruent conditions. Fillers are commonly used to obscure the nature of the task^84–87^. In the congruent condition, the number of words was consistent with the word’s meaning, such as ‘‘one’’, ‘‘two’’, ‘‘three’’, or ‘‘four”, but not in the incongruent condition. Participants were required to report the number of words (1–4) on the screen via button pressing, regardless of word meaning. The task consisted of three sessions, lasting approximately 8 min each. Within each testing session, trials were presented in a pseudorandomized order such that no more than three succeeding trials were from the same condition^88^. Each trial began with a fixation (e.g., white square) for 150 ms, followed by an emotional prime of a neutral, filler, or pandemic-related picture presented for 250 ms. After the emotional primes, an item of the counting Stroop task was presented for 1500 ms. The intertrial intervals (ITIs) were varied randomly among 1000 ms, 1500 ms, and 2000 ms (see Fig. 1). The inclusion of a variable ITI is designed to prevent the carry-over effects of emotional responses^82,83,88,89^. Moreover, the pseudorandomized presentation of the trials should ensure that any carryover would fall equally on emotional and neutral trials^90^. In the present study, reaction time (RT) was used as the behavioral index of Stroop performance. Longer response latencies are interpreted as greater interference and higher attentional bias to the emotional content of the presented stimuli^91–93^. Analyses for RT were performed on only correct trials, and we removed outlier RTs more than 2.5 SDs from the mean in each condition for each participant. After removing outliers, the mean RTs were calculated within each condition. In total, 4.19% of observations were excluded. Subsequently, attentional bias was calculated by subtracting the neutral trials’ RT from the pandemic trials’ RT in the incongruent condition^82,83^.

#### Stimuli characteristics

As stimuli, 61 pictures were selected from the International Affective Picture System (IAPS)^94^, and 29 pictures were chosen from the public photo archives^95–99^; each picture was presented twice in the task, once each in the congruent and incongruent conditions. Pictures were matched for size and brightness. Twenty-nine adults who did not participate in the experiment were asked to rate the emotional characteristics of the stimuli on a 9-point scale, including emotional valence (1 = very unpleasant to 9 = very pleasant) and arousal (1 = very calm to 9 = highly arousing)^82,100^. Additionally, participants were asked to rate whether the stimuli were associated with COVID-19 on a 9-point scale (1 = not at all to 9 = strongly associated). Pandemic-related pictures had values of 5 or higher on the relatedness scale. Table 1 lists the stimuli characteristics for each condition. Regarding emotional characteristics, valence was significantly lower and arousal was significantly higher for pandemic-related pictures than for neutral pictures (*p*s < 0.05). Moreover, the selected pandemic-related pictures had a higher pandemic-relatedness score than the neutral pictures (*p* < 0.05).

**Table 1:** Stimuli characteristics for each condition.

Condition	Valence		Arousal		COVID-19 relatedness	
	*M*	*SD*	*M*	*SD*	*M*	*SD*
Pandemic	3.01	0.43	5.51	0.44	7.26	0.56
Neutral	5.41	0.64	3.56	0.40	1.55	0.29
Filler	2.53	0.71	6.67	0.52	1.65	0.46

### Data analysis

All analyses were performed using SPSS version 25.0. First, descriptive statistics and intercorrelations among all variables were calculated. Then, to test the hypotheses, two moderation models were constructed using Hayes PROCESS macro-Model 1^101^. To examine the first aim of the study, we entered alexithymia as the moderator of the relationship between pandemic-related stress and state anxiety of individuals. To examine the second aim, we entered alexithymia as the moderator of the relationship between pandemic-related stress and attentional bias. In the analysis, both alexithymia and pandemic-related stress were centered to the mean, and the bias-corrected bootstrap 95% confidence interval, based on 5000 bootstrap samples, was calculated to evaluate the conditional effect at different levels of alexithymia^101^. Using the Johnson–Neyman method, we plotted the conditional effects of these two models.

## Results

### Descriptive statistics

Descriptive statistics and Pearson’s correlation coefficients among all variables are shown in Table 2.

**Table 2:** Descriptive statistics and intercorrelations among all variables.

Variable	1	2	3	4
1. Alexithymia				
2. Pandemic-related stress	0.16			
3. State anxiety	0.28**	0.22*		
4. Attentional bias	− 0.17	0.35***	0.13	
*M*	45.73	13.39	38.47	− 4.10
*SD*	10.11	5.98	9.72	30.15

N = 103; *M* mean, *SD* standard deviation.

*p < 0.05, **p < 0.01, ***p < 0.001.

### Alexithymia, pandemic-related stress, and state anxiety

**Figure 2:**
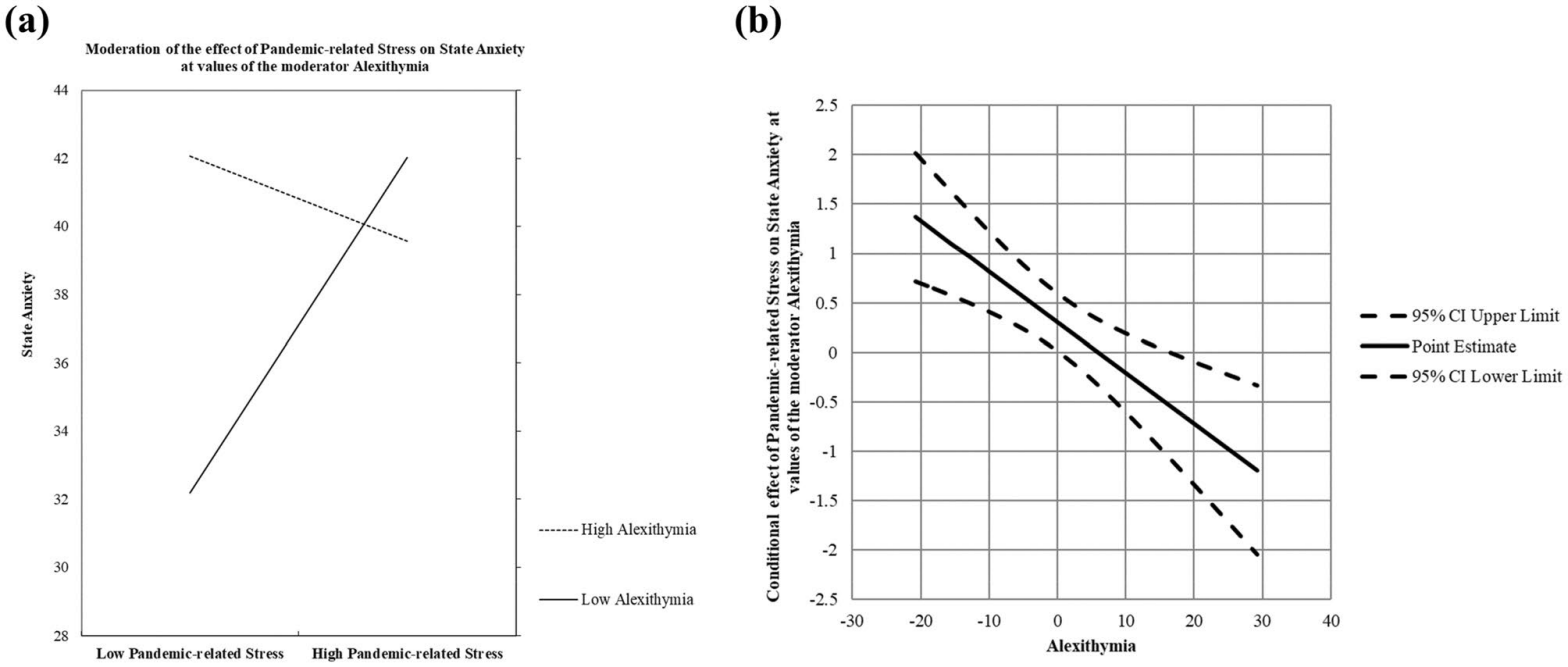
The conditional effect of pandemic-related stress on state anxiety at different levels of alexithymia.

As shown in Table 3 (Model 1), three predictors accounted for 21% of the total variance in the outcome, *F* (3, 99) = 8.96, *p* < 0.001. Regarding the main effects, the results revealed that greater pandemic-related stress was significantly associated with greater state anxiety, *b* = 0.31, *SE* = 0.15, *t* = 2.09, *p* < 0.05, and that higher alexithymia levels were significantly associated with greater state anxiety, *b* = 0.18, *SE* = 0.09, *t* = 2.08, *p* < 0.05. Additionally, the pandemic-related stress × alexithymia interaction was significant, *b* = − 0.05, *SE* = 0.01, *t* = − 3.66, *p* < 0.001, and ΔR^2^ = 0.11, indicating that alexithymia moderated the association between pandemic-related stress and state anxiety. The interaction is plotted in Fig. 2A. The conditional effect of pandemic-related stress on state anxiety was calculated at 1 *SD* below the mean, the mean, and 1 *SD* above the mean of alexithymia. The results revealed that in participants with lower levels of alexithymia, greater pandemic-related stress caused greater state anxiety, *b* = 0.82, *SE* = 0.21, *t* = 4.00, *p* < 0.001. However, with increased levels of alexithymia, the effect of pandemic-related stress on state anxiety became weaker. Furthermore, application of the Johnson–Neyman method revealed that the effect of pandemic-related stress on state anxiety was nonsignificant when the alexithymia level of participants was higher than 46.05 (see Fig. 2B).

**Table 3:** Moderation models.

Outcome	Model 1		Model 2	
	State anxiety		Attentional bias	
Predictor	*b*	*t*	*b*	*t*
PS	0.31	2.09*	2.00	4.40***
ALEX	0.18	2.08*	− 0.81	− 2.95**
PS × ALEX	− 0.05	− 3.66***	− 0.09	− 2.06*
Model R^2^	0.21, *F* (3, 99) = 8.96***		0.22, *F* (3, 99) = 9.13***	

*PS* pandemic-related stress, *ALEX* alexithymia.

*p < 0.05 **p < 0.01 ***p < 0.001.

### Alexithymia, pandemic-related stress, and attentional bias

**Figure 3:**
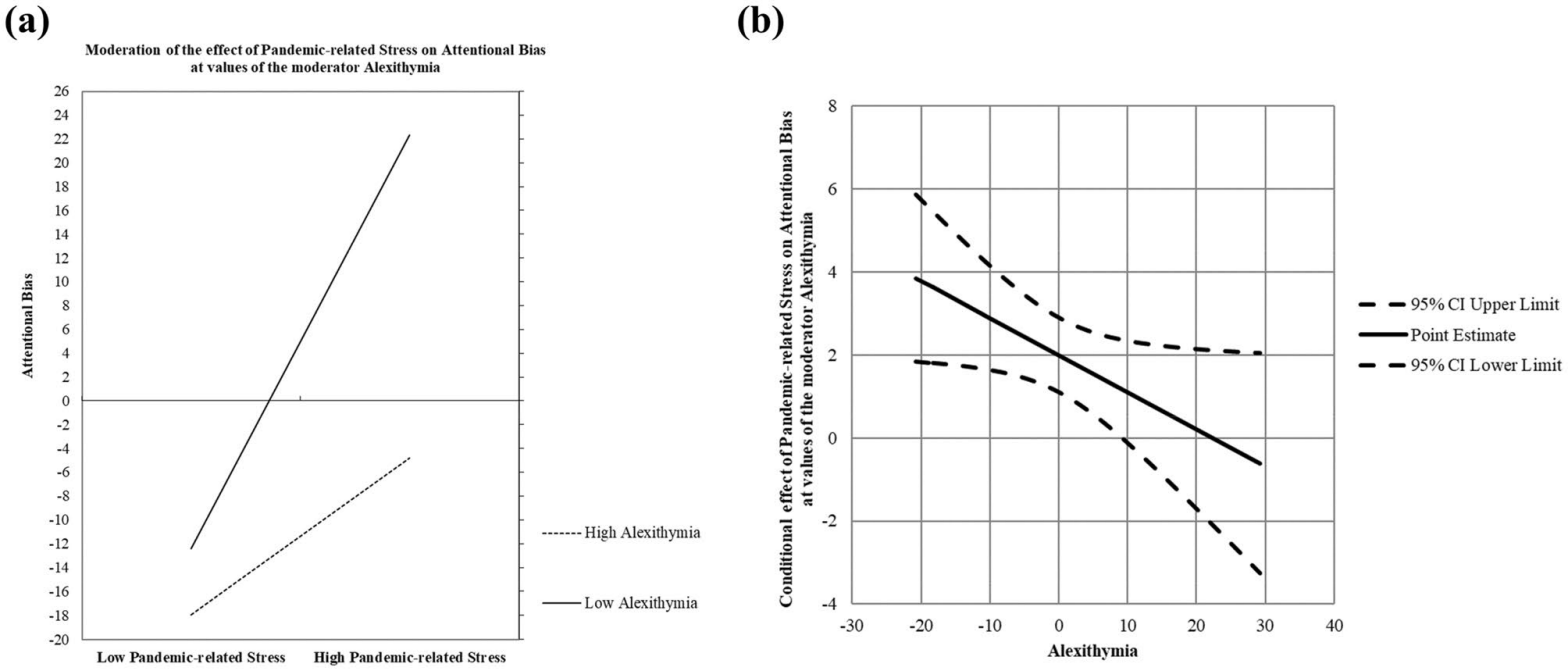
The conditional effect of pandemic-related stress on attentional bias at different levels of alexithymia.

As shown in Table 3 (Model 2), three predictors accounted for 22% of the total variance in the outcome, *F* (3, 99) = 9.13, *p* < 0.001. Regarding the main effects, the results revealed that greater pandemic-related stress was significantly associated with greater attentional bias, *b* = 2.00, *SE* = 0.46, *t* = 4.40, *p* < 0.001, and that higher levels of alexithymia were significantly associated with lower attentional bias, *b* = − 0.81, *SE* = 0.27, *t* = − 2.95, *p* < 0.01. Additionally, the pandemic-related stress × alexithymia interaction was significant, *b* = − 0.09, *SE* = 0.04, *t* = − 2.06, *p* < 0.05, and ΔR^2^ = 0.03, indicating that alexithymia moderated the association between pandemic-related stress and attentional bias. The interaction is plotted in Fig. 3A. The conditional effect of pandemic-related stress on attentional bias was also calculated at 1 *SD* below the mean, the mean, and 1 *SD* above the mean of alexithymia. The results revealed that in participants with lower levels of alexithymia, greater pandemic-related stress caused greater attentional bias, *b* = 2.91, *SE* = 0.64, *t* = 4.53, *p* < 0.001. However, with increased levels of alexithymia, the relationship between pandemic-related stress and attentional bias became weaker. Furthermore, application of the Johnson–Neyman method revealed that the effect of pandemic-related stress on attentional bias was nonsignificant when the alexithymia level of participants was higher than 54.90 (see Fig. 3B).

## Discussion

This study aimed to determine whether alexithymia contributed to vulnerability to anxiety levels in response to COVID-19-related stressors among Taiwanese college students during the early phase of the Omicron wave in late spring of 2022. Our results revealed that alexithymia significantly moderated the relationship between pandemic-related stressors and levels of anxiety. Moreover, we found that among individuals with a higher level of alexithymia, exposure to these stressors had a lesser impact on anxiety levels. Our second aim was to explore the cognitive mechanism of attentional bias in response to stressful events during the pandemic. Our results indicate that alexithymia moderated the association between pandemic-related stressors and attentional bias. For individuals with higher alexithymia levels, exposure to the stressors had a smaller impact on attentional bias. Currently, our findings have several theoretical implications in the context of the COVID-19 pandemic.

Our first finding was that the degree of exposure to pandemic-related stressors was associated with anxiety levels and that this relationship was moderated by alexithymia. The stress-alexithymia hypothesis predicts that in individuals with higher levels of alexithymia, higher arousal leads to greater mental problems^34^. Interestingly, in our cross-sectional study during the Omicron wave of the pandemic, pandemic-related stress had a lesser impact on anxiety among individuals with higher levels of alexithymia. Our results could be interpreted as avoidance of COVID-19-related stressors by alexithymic individuals. According to the accounts of experiential avoidance, alexithymia is considered a learned tendency to use maladaptive coping to deal with severe stress^102,103^. This tendency in individuals with alexithymia may be related to an unwillingness to tolerate aversive sensations, emotions, thoughts and memories, leading to alterations of the form or frequency with which they confront them^33,104^. Additionally, this learned tendency is reinforced and maintained as it provides immediate relief and reduced distress in threatening situations^33,105,106^; however, it is ultimately associated with the accumulation and intensification of unwanted emotions^107^. Thus, this detrimental effect of temporary relief from stress in individuals with alexithymia may predict the development of psychopathology^104,108–110^. The present results further suggest that alexithymia is a factor moderating mental health outcomes in the context of exposure to pandemic-related stressors, and these early defensive reactions to avoid stress may accumulate unpleasant emotions over time.

Our second finding was that alexithymia moderates the association between exposure to pandemic-related stressors and attentional bias. These findings are consistent with the vigilance-avoidance hypothesis of attentional bias^64^, which suggests that among individuals with higher exposure to pandemic-related stressors, higher levels of alexithymia indicate less attention toward negative information compared to neutral information (i.e., attentional bias oriented away from threatening information). The attentional bias literature has proposed an underlying mechanism of attentional allocation for individuals with alexithymia^111,112^. Specifically, decisions regarding coping with unwanted emotions are thought to be determined by appraisals of emotions (i.e., evaluation of whether the emotion is beneficial or detrimental to current goals and thus initiating up- or downregulation of emotions)^112,113^. With this mechanism, potential threats are detected in a way that buffers the anticipated harm^45,55^. In the present study, when individuals with alexithymia encountered COVID-19-related stimuli, the defensive pattern of early vigilance followed by avoidance prevented constant contact with threatening information (e.g., increasing numbers of confirmed cases and case fatality rates); thus, negative affect did not interfere with subsequent attentional processing (i.e., less attentional depletion) during the later decision stage. Importantly, early detection of a threat and subsequent shifting of attention away from the threat may provide temporary relief from stressors^114–116^. However, using this defensive mechanism is likely to exacerbate psychopathological problems in the long term^117,118^. In such a setting, the use of attentional avoidance of threatening information (or less problem-focused coping) makes individuals with alexithymia more vulnerable to increased exposure to pandemic-related stressors over time.

Taken together, the current findings have implications concerning for the mental wellbeing of individuals during the pandemic. Even though the college students in this study were a “low-risk” population (i.e., not predicted to suffer from psychiatric disorders at the early phase of the pandemic), individuals with alexithymia may have used maladaptive coping to downregulate emotions and achieve immediate relief from distress. However, their negative emotions may gradually accumulate during the postpandemic period. Our findings that alexithymia had a moderating role in this stress-symptom relationship may imply that alexithymic traits may identify vulnerable individuals under pandemic conditions. Additionally, this study further found that individuals with alexithymia may give limited attention to essential COVID-19-related information. Therefore, as the pandemic continues, these findings emphasize the importance of implementing policies to alleviate the stress (e.g., by providing support for financial loss, fears of contagion, and social isolation) experienced by young adults. These procedures may help to increase feelings of certainty and control, thus reducing tensions and improving resilience to stress during the pandemic. Moreover, it is plausible that the attentional avoidance of threats may stem from uncertainty; therefore, facilitating the absorption of accurate knowledge about COVID-19 (e.g., symptoms and characteristics of Omicron variants, preventive procedures) and conducting interventions that emphasize improving attentional control (e.g., mindfulness training, attentional bias modification) may potentially eliminate uncertainty^119–122^, facilitate the interpretation of threatening information, and enable better coping in the face of environmental threats^123^.

This study provides an integrated framework for exploring the role of alexithymia in mental health during the COVID-19 pandemic. However, certain limitations should be considered. First, the cross-sectional design means that conclusions regarding how alexithymia modulates the relationship between exposure to stressors and anxiety levels during the pandemic should be interpreted with caution^75,124^. Future longitudinal studies are needed to further examine whether alexithymia moderates the relationship between the perceived stress of pandemic events and changes in psychological symptoms over time. Second, the use of self-report measures (such as exposure to COVID-19-related stressors, alexithymia, and anxiety level) may lead to report bias; thus, other objective measurements should be considered in the future. Third, although the interpretations of attentional bias are based on a single, well-established measurement that shows consistent association with psychopathology^92,125^, alternative measurements (e.g., the Posner cueing task, dot-probe task accompanied by the use of eye-tracking technology) should be considered in the future for further validation. Fourth, our findings may not be generalized to the entire population due to the exclusion criterion (i.e., the anxiety and depressive symptoms). Considering that the effects of stress during the pandemic and alexithymia on mental health were found in relatively low-risk students in the current study, it is reasonable to believe that these findings may not be generalizable to populations with higher risk. Fifth, the relationship between attentional bias and mental disorders (i.e., anxiety) may be bidirectional, with anxiety exacerbating attentional bias and attentional bias aggravating anxiety^126^. Here, we examined whether alexithymic traits elicited attentional bias toward threat-related information. Future studies should further explore whether the maladaptive attentional bias in individuals with alexithymia lead to long-term mental health problems. Finally, recent research has reported that increasing numbers of psychiatric symptoms, such as anxiety and sleep disturbance, developed or persisted after acute COVID-19^127^. The severity of sleep disturbance was closely linked to high levels of anxiety and stress, reducing quality of life^128^. Alexithymia may also modulate other mental health issues during the pandemic, such as sleep disturbance and anxiety disorders; hence, further studies should clarify the role of alexithymia in the relationship between pandemic-related stressors and various mental health problems to develop a more comprehensive understanding of the long-term impacts of COVID-19.

In conclusion, pandemic-related stress had a lesser impact on anxiety among individuals with higher levels of alexithymia. Our results support experiential avoidance; in other words, that individuals with alexithymia may avoid unfavorable thoughts and emotions (e.g., stressful experiences). We further demonstrated that among individuals with higher perceived psychological stress, higher levels of alexithymia indicated less attentional bias toward COVID-19-related stimuli. This avoidance may provide immediate relief and decreased levels of anxiety during the COVID-19 pandemic, but this inflexible coping strategy may increase the risk for mental symptoms in reaction to pandemic-related stressors in the long term. Thus, future research should consider the long-term influences of the COVID-19 pandemic on psychopathology.

## Data Availability

The data and material used and/or analyzed during the current study are available from the corresponding authors (SHL and KTL) on reasonable request.
